# Do We Have a Common Understanding of How Vaccine Policy Affects Health Equity? Evaluating Variability in the Advisory Committee on Immunization Practices’ Equity Assessment

**DOI:** 10.3390/vaccines13030214

**Published:** 2025-02-21

**Authors:** Kathleen Dooling, Elif Alyanak, Dial Hewlett, Haley Payne, Vincenza Snow, Mitchell Finkel, Maura Burns, Brett Hauber, Joshua Coulter, Ronika Alexander-Parrish

**Affiliations:** 1 Pfizer, Collegeville, PA 19426, USA; 2 Avalere Health, Washington, DC 20005, USA; 3 Westchester County Department of Health, White Plains, NY 10601, USA

**Keywords:** ACIP, EtR, equity, evidence, recommendations, policy

## Abstract

Background/Objectives: The Advisory Committee on Immunization Practices’ (ACIP) Evidence to Recommendation (EtR) Framework has assessed vaccine equity, in addition to clinical and epidemiological data, since 2020. The domain’s use has not yet been evaluated. Methods: Authors assessed web-published, Equity domain-inclusive ACIP Work Group EtR presentations occurring between October 2020 and October 2023. Domain judgments were scored and assigned variability ratings based on the number and spread of domain categories selected. Equity domain trends were evaluated using sample statistics and one- and two-way analyses of variance. Results: Of the 44 assessed EtRs, 27 (61.4%) had variable judgments for at least one domain; 9 (20.4%) had variable Equity judgments. Across domains, Values had the greatest variability, followed by Equity. Across disease targets, EtRs assessing products for RSV prevention were most variable. Pediatric product EtRs had greater variability than adult products, and EtRs resulting in shared clinical decision-making (SCDM) recommendations had greater variability than those resulting in routine recommendations. Conclusions: Values and Equity domains judgment imprecision highlights a need for additional clarity to support consistent assessment.

## 1. Introduction

The Advisory Committee on Immunization Practices (ACIP) is the federal immunization technical advisory group for the US responsible for developing recommendations on the use of immunizations [1,2]. Recommendation development involves systematic evidence review and appraisal from experts and disease-specific Work Groups (WGs) followed by a final ACIP vote. WGs are subgroups of the ACIP “responsible for collection, analysis, and preparation of information for presentation, discussion, deliberation, and vote by the ACIP in an open public forum” [3]. WGs are composed of at least two ACIP voting members, liaison organization representatives, expert consultants, consumer representatives, and federal ex-officio members.

To support the ACIP’s efforts toward process transparency and consistency, in 2018, the ACIP adopted the Evidence to Recommendation (EtR) framework with six domains: Public Health Problem, Benefits and Harms, Values, Acceptability, Feasibility, and Resource Use [4]. For each policy question that the ACIP considers when making a vaccine use recommendation, WGs assess vaccine evidence against criteria outlined for each EtR domain. These domain-specific assessments lead to a WG judgment. WGs will present EtR evidence assessments and related judgments (“EtR Presentations”) to the full ACIP during public meetings, which, in turn, inform ACIP recommendations.

In 2020, the ACIP adopted a seventh EtR domain focused on health equity (“Equity”) in response to the disproportionate impact of COVID-19 on vulnerable populations [5,6]. The Equity domain encourages the ACIP to consider the impact of a proposed vaccine recommendation on health equity. Similar to the other EtR domains, WGs will assess vaccine evidence against specific criteria for Equity and make a judgment. Paula Braveman (2022) states, “health equity means that everyone has a fair and just opportunity to be as healthy as possible” [7]. The EtR User’s Guide calls for assessing equity as an outcome only of vaccination; specifically, identifying disadvantaged groups related to preventable disease burden and a potential recommendation [5]. Use of this domain in the 4 years since its inception could provide insight into potential improvements to support a shared understanding of equity across the ACIP.

To evaluate the degree of shared understanding of the Equity domain among ACIP WGs, a retrospective analysis of each EtR presentation given to the ACIP by WGs between October 2020 and October 2023 was conducted to determine how much WG judgment variability existed for the Equity domain compared to the other EtR domains. For the purposes of this research, ‘variability’ is defined as the degree of consensus among WG members around a single judgment; for example, the Equity domain of the EtR framework aims to understand to what extent the intervention will impact health equity (i.e., “reduce”, “probably reduce”, “probably no impact”, “probably increase”, “increase”, “varies”, “don’t know”). Variability, in this case, would be reflected as two or more options chosen from this list, rather than a single option agreed to by all WG members. Quantification of this variability is further described in Materials and Methods.

## 2. Materials and Methods

### 2.1. Data Collection

This retrospective analysis examined all WG EtR presentations given during ACIP meetings and published to the ACIP’s public repository between October 2020 (when the Equity domain was first implemented) and October 2023 (the most recent EtR presentations available at the time of study design) [8]. EtR presentations that occurred outside of this time frame and presentations that did not include an Equity domain judgment were excluded from the analysis. While all ACIP recommendations include final EtR judgments in the Centers for Disease Control and Prevention (CDC’s) *Morbidity and Mortality Weekly Report*, the published iteration includes additional considerations raised during deliberations rather than the WG’s final judgment; as such, only presented judgments were included in the dataset.

For each EtR presentation, researchers collected the WG judgment(s) for each EtR domain, disease target(s) of the products being assessed, the age group of the population being considered for vaccination, and the final ACIP recommendation type (i.e., routine, risk-based, age-based, shared clinical decision-making [SCDM]). Additional researchers then reviewed the initial data collection and cross-walked each EtR presentation to the ACIP’s List of EtR Framework Tables [9].

### 2.2. Quantitative Scoring

EtR domains are assessed using categorical variables to signify whether WG members believe the intervention will or will not meet domain criteria, as outlined in the User’s Guide [4]. Specifically, as noted above, the Equity domain aims to understand to what extent the intervention will impact health equity. For each domain, researchers collated WG judgments and quantified the degree of variability among WG member judgments using the following scoring rubric to support statistical testing:

- **0 = None:** 1 option chosen;
- **1 = Low:** 2 options chosen; options are next to each other in scale;
- **2 = Moderate:** 2 options chosen with at least 1 degree of separation; both options within positive or within negative judgments, inclusive of ‘probably no impact’;
- **3 = High:** 3 to 5 options selected except for ‘varies’ or ‘don’t know’ **or** 2 options with at least 1 degree of separation across positive and negative judgments;
- **4 = Very High:** All options selected **or** ‘varies’ or ‘don’t know’ selected **or** 2 options with at least 2 degrees of separation across positive and negative judgments.

### 2.3. Statistical Analyses

Once each WG judgment was assigned a quantitative score, these scores were used to first characterize the magnitude of variability across all WG EtR presentations within the Equity domain. The magnitude of Equity domain variability was then characterized against the magnitude of variability for all other EtR domains. Sample statistics (i.e., mean, median, standard deviation, and range) were provided for all judgments across domains and were assigned to one of the above variability categories. To assess the statistical significance of the difference between Equity domain judgment variability compared to variability for the other EtR domains, a one-way analysis of variance (ANOVA) was conducted. To assess the statistical significance of the difference between Equity domain judgment variability compared to variability for the other EtR domains by disease target, recommended age group, and ACIP recommendation type, a two-way ANOVA was conducted. Statistical significance was assessed at the 10% level.

## 3. Results

Between October 2020 and October 2023, 52 EtRs were presented; 8 lacked an Equity domain judgment and were excluded, resulting in 44 EtRs for analysis inclusion and a total of 290 domain judgments. Most judgments (*n* = 240; 82.8%) indicated consensus, meaning WG members made the same selection in their domain assessment.

At the EtR level, 27 (61.4%) had variable judgments for at least one domain; most of these judgments had low variability (*n* = 32; 64%) followed by very high variability (*n* = 16; 32%). All domains had a median variability score of 0, and all except the Public Health Problem domain had variability scores ranging from 0 to 4 (Public Health Problem demonstrated no variability across all assessed EtRs).

Within the Equity domain, 22 judgments (50%) indicated the reviewed product would increase equity, while the other 22 (50%) indicated reduced equity or a lack of clarity. Most Equity domain judgments had no variability (*n* = 35; 79.6%), four judgments (9.1%) had low variability, two judgments (4.5%) had high variability, and three judgments (6.8%) had very high variability.

Overall, Values domain judgments had the greatest average variability (0.6) followed by Equity (0.5). Mean variability scores for all domains were less than one (“Low”) and most judgments were more than one standard deviation from the mean. There was at least one ‘very high’ variability score in most domains; this did not occur for any judgments within the Public Health Problem domain, for which there was complete consensus (variability score = 0). Summary statistics for all domains can be found in Table 1, and the full range of variability scores by domain for each EtR can be found in Supplemental Table S1.

### Equity Variability by Disease Target, Age Group, and Recommendation Type

Among all disease targets, EtRs assessing RSV (*n* = 6), COVID-19 (*n* = 9), and pneumococcal (*n* = 12) vaccines accounted for most Equity domain variability. EtRs assessing RSV products had the greatest mean Equity judgment variability (1.2), followed by EtRs assessing COVID-19 (0.4) and pneumococcal (0.3) vaccines.

There were 29 EtRs assessing products for adults (≥18 years) and 11 for children (<18 years). EtRs assessing pediatric products had higher mean Equity domain variability (0.7) than those assessing adult products (0.4), though both were considered low.

Finally, across recommendation types, 36 EtRs resulted in routine recommendations while 8 EtRs resulted in shared clinical decision-making (SCDM) recommendations. Mean Equity variability for EtRs that led to routine recommendations was found to be lower than for EtRs that led to SCDM recommendations (0.3 vs. 1.5, respectively).

Mean variability stratified by disease target, age group, and recommendation type across all EtR domains can be found in Table 2. Mean, median, and the range of variability for the Equity domain stratified by disease target, age group, and recommendation type can be found in Table 3.

Overall observed differences in variability between EtR domains were statistically significant (*p* = 0.094). Differences in mean domain variability by recommendation type were also statistically significant (*p* = 0.068). Differences in mean domain variability by disease target and age group were not statistically significant (*p* = 0.363 and *p* = 0.401, respectively).

## 4. Discussion

### 4.1. Values and Equity Domains Demonstrated the Greatest Variability

In this analysis, WG judgments for the Values and Equity EtR domains demonstrated the greatest mean variability (0.6 and 0.5, respectively). While Equity domain variability aligned with the original hypothesis, the judgment variability demonstrated within the Values domain introduced an additional insight.

The 2024 ACIP Handbook for Developing Evidence-Based Recommendations provides systematic review instruction and guides evidence certainty appraisal; however, details on qualitative data extraction and review are limited [10]. Similarly, the EtR User’s Guide Values and Equity guidance is brief: “Work groups may conduct a high-level search to identify any publications reporting on issues of health equity or inequity… If no evidence is identified, then the work group should provide additional considerations… to the best of their ability” [4]. These insights may present opportunities to improve ACIP Values and Equity review and further develop guidance for evidence interpretation.

Disease prevalence evidence is often limited to individuals with access to quality care and an accurate diagnosis, potentially excluding those without adequate care access and/or quality [11]. This exclusion may challenge the establishment of a baseline from which ACIP members can determine the equity impact of a vaccine. For the stakeholders responsible for generating evidence for WG consideration, this could present a unique opportunity to establish and apply population and data standards fit for purpose to conduct equity and patient experience research and to establish tools to predict the effect of a vaccine policy on equity.

WGs assess platform coverage trends, as available. In the event of a novel platform, the lack of a standard metric or threshold may introduce Equity judgment uncertainty. Additionally, existing platform trends may not reflect the equity impact of a new vaccine. Finally, equity data across various demographics may be inconsistent across studies. Addressing these challenges could begin with a clear consensus definition of equity. Plamondon and Shahram (2024) suggest defining equity in three ways: (1) as a concept, (2) as a specific outcome, and (3) as an individual experience [12]. The current approach is limited in its applicability to equity as a concept and an individual experience; as such, a consensus definition could inform the scope of evidence assessed, including analysis types, to understand the impact of a vaccine policy on equity.

The Feasibility EtR domain, which focuses on the ease of implementing a new vaccine, contains a checklist to guide WG and ACIP member assessments of evidence-based considerations and weighing of different options. Similar tools for Values and Equity could leverage validated frameworks like the Health Equity Implementation Framework, the World Health Organization’s Health Equity Assessment Tool, or the Avalere/FasterCures Patient-Perspective Value Framework, tailored to Committee- and vaccine-specific evidence needs [13,14,15]. For example, during its review of older adult RSV vaccines, WG members noted that RSV-related hospitalizations varied across adult demographics, indicating a need to present and understand stratified evidence of the impact of RSV vaccination on hospitalization [16]. Standardized frameworks may guide evidence generation to ensure their availability for ACIP review and recommendation.

### 4.2. Equity Judgment Variability Emerged as Associated with SCDM Recommendations

The greatest cross-category difference in Equity domain judgment variability was between EtRs resulting in SCDM (1.5) vs. routine use recommendations (0.3). This association was found to be statistically significant (*p* = 0.068). Various stakeholders have raised concerns regarding SCDM recommendations and subsequent vaccine implementation barriers [17,18]. Reducing Equity domain uncertainty, combined with additional study of the association between uncertainty and recommendation type, could further address these concerns.

### 4.3. Equity Domain Evaluation Limitations

There were several key limitations to this analysis. First, the sample size was limited to the 44 EtR frameworks presented between October 2020 and October 2023 that included Equity domain judgments. This small sample size limited the ability to stratify the analysis and to power statistical analysis to *p* = 0.05. Further, other factors not assessed, including product characteristics, breadth of evidence included in each review, collective and individual WG member dynamics, and external societal sentiments and the material environment, may have influenced variability. Findings are also unique to the ACIP and may not be generalizable to National Immunization Technical Advisory Groups in other countries. Finally, this analysis did not compare the types of evidence submitted for each EtR, which may have varied from submission to submission and could have informed WG judgment decisions.

## 5. Conclusions

While the research question sought to understand variability in Equity judgments, we found that Values judgments demonstrated similar variability, suggesting opportunities for additional resources to support consistent review and judgment.

As the ACIP endeavors to optimize vaccine recommendations for adults and children, opportunities to innovate in the execution of the review process could include convening vaccine ethics and health equity specialists to inform an updated evidence review framework. Further, the ACIP, facilitated by a liaison organization, could expand WG membership to equity and social science experts who are also knowledgeable in vaccines.

A shared understanding of equity, including inequity determinants and drivers, can support refining Equity domain assessments, particularly when evaluated against the conditions potentially created by a vaccine policy under consideration. Recent policy proposals like the Vaccines for Adults program could also affect the equity landscape upon which the ACIP makes recommendations. Finally, given the ACIP’s longstanding and lauded role in advising CDC vaccine policy, ensuring Committee decision-making is understood by the general public may support its sustainability, particularly as vaccine confidence remains a public health priority.

## Figures and Tables

**Table 1:** Summary statistics—EtR domain variability ^1^ for all recommendations (*n* = 44) ^2^.

Category	Mean (SD)	Range
Values	0.6 (1.3)	0–4
Equity	0.5 (1.1)	0–4
Resource Use	0.5 (1.1)	0–4
Acceptability	0.4 (0.9)	0–4
Benefits and Harms	0.3 (1.0)	0–4
Feasibility	0.2 (0.7)	0–4
Public Health Problem	0.0 (0.0)	0–0

^1^ Variability was measured on a scale from 0 (none) to 4 (very high). ^2^ The median and mode were zero (0) for all categories.

**Table 2:** Mean (SD) EtR domain variability ^1^ stratification by disease target, age group, and recommendation type.

Category	Values	Equity	Resource Use	Acceptability	Benefits and Harms	Feasibility	Public Health Problem
**Disease Target**							
COVID-19 (*n* = 9)	1.3 (2.0)	0.4 (1.3)	0.0 (0.0)	0.0 (0.0)	0.0 (0.0)	0.0 (0.0)	0.0 (0.0)
Hepatitis B (*n* = 2)	0.0 (0.0)	0.0 (0.0)	0.0 (0.0)	1.0 (N/A)	0.0 (0.0)	0.0 (0.0)	0.0 (0.0)
Herpes Zoster (*n* = 2)	0.0 (0.0)	0.0 (0.0)	0.0 (0.0)	0.0 (0.0)	0.0 (0.0)	0.0 (0.0)	0.0 (0.0)
Influenza (*n* = 2)	2.0 (2.8)	0.0 (0.0)	0.0 (0.0)	1.0 (0.7)	0.0 (0.0)	0.0 (0.0)	0.0 (0.0)
Mpox (*n* = 5)	0.0 (0.0)	0.0 (0.0)	0.8 (1.8)	0.0 (0.0)	0.8 (1.8)	0.8 (1.8)	0.0 (0.0)
Pneumococcal (*n* = 12)	0.3 (0.5)	0.3 (0.5)	0.6 (1.2)	0.8 (1.5)	0.2 (0.4)	0.0 (0.0)	0.0 (0.0)
Rabies (*n* = 2)	0.0 (0.0)	0.0 (0.0)	0.0 (0.0)	0.0 (0.0)	0.0 (0.0)	0.0 (0.0)	N/A (N/A)
RSV (*n* = 6)	0.5 (0.6)	1.2 (1.5)	0.3 (0.5)	1.0 (0.0)	1.3 (2.1)	0.5 (0.6)	0.0 (0.0)
**Age Group**							
Adult (*n* = 29)	0.3 (0.8)	0.4 (0.8)	0.4 (1.1)	0.5 (1.1)	0.4 (1.2)	0.3 (0.8)	0.0 (0.0)
Pediatric (*n* = 11)	1.4 (1.8)	0.7 (1.6)	0.7 (1.2)	0.3 (0.5)	0.2 (0.4)	0.0 (0.0)	0.0 (0.0)
**Recommendation Type**							
Routine (*n* = 36)	0.5 (1.3)	0.3 (0.9)	0.3 (0.8)	0.4 (1.0)	0.3 (1.0)	0.1 (0.7)	0.0 (0.0)
SCDM (*n* = 8)	0.5 (1.4)	1.5 (1.6)	0.8 (1.4)	0.6 (0.5)	0.5 (1.4)	0.5 (1.4)	0.0 (0.0)

^1^ Variability was measured on a scale from 0 (none) to 4 (very high).

**Table 3:** Summary statistics—equity domain variability ^1^ stratification by disease target, age group, and recommendation type ^2^.

Category	Mean (SD)	Median	Range
Disease Target			
COVID-19 (*n* = 9)	0.4 (1.3)	0.0	0–4
Hepatitis B (*n* = 2)	0.0 (0.0)	0.0	0–0
Herpes Zoster (*n* = 2)	0.0 (0.0)	0.0	0–0
Influenza (*n* = 2)	0.0 (0.0)	0.0	0–0
Mpox (*n* = 5)	0.0 (0.0)	0.0	0–0
Pneumococcal (*n* = 12)	0.3 (0.5)	0.0	0–1
Rabies (*n* = 2)	0.0 (0.0)	0.0	0–0
RSV (*n* = 6)	1.2 (1.5)	0.5	0–3
Adult (*n* = 29)	0.4 (0.8)	0.0	0–3
Pediatric (*n* = 11)	0.7 (1.6)	0.0	0–4
Routine (*n* = 36)	0.3 (0.9)	0.0	0–4
SCDM (*n* = 8)	1.5 (1.6)	1.0	0–4

^1^ Variability was measured on a scale from 0 (none) to 4 (very high). ^2^ The mode was zero (0) for all categories.

## Data Availability

The original contributions presented in the study are included in the article/Supplementary Materials. Further inquiries can be directed to the corresponding author.

## Supplementary Materials

The following supporting information can be downloaded at https://www.mdpi.com/article/10.3390/vaccines13030214/s1: Table S1: Variability scores across EtR domains.
