# Supplementary material for: Do We Have a Common Understanding of How Vaccine Policy Affects Health Equity? Evaluating Variability in the Advisory Committee on Immunization Practices’ Equity Assessment
Source: Vaccines (Basel). 2025 Feb 21;13(3):214. doi: 10.3390/vaccines13030214 (PMC11946652; doi:10.3390/vaccines13030214)
Supplement: Supplementary file 1 [file vaccines-13-00214-s001.zip › vaccines-3266377-supplementary.pdf]

Table S1. Variability[1] Scores Across EtR Domains

EtR Presentation Date	EtR	Pathogen/Disease Area	Product	Domain-Specific Variability Scores						
				Equity	Public Health Problem	Benefits & Harms	Values	Acceptability	Feasibility	Resource Use
Dec. 12, 2020	Use of Pfizer-BioNTech COVID-19 Vaccine under EUA for individuals aged 16 years and older	COVID-19	Comirnaty	**0**	**0**	**0**	**0**	**0**	**0**	**0**
Dec. 19, 2020	Use of Moderna COVID-19 Vaccine under EUA for individuals aged 18 years and older	COVID-19	SPIKEVAX	**0**	**0**	**0**	**0**	**0**	**0**	**0**
Feb. 28, 2021	Use of Janssen COVID-19 Vaccine under EUA for individuals aged 18 years and older	COVID-19	Jcovden	**0**	**0**	**0**	**0**	**0**	**0**	**0**
Jun. 24, 2021	Dengvaxia administration to individuals aged 9-16 years with laboratory-confirmed previous dengue infection living in endemic areas	Dengue	DENGVAXIA	**0**	**0**	**0**	**0**	**0**	**0**	**1**
Jun. 24, 2021	A booster dose of rabies vaccine after the 2-dose PrEP schedule for individuals at elevated risk of recognized exposures	Rabies	IMOVAX RabAvert	**0**	**N/A**	**0**	**0**	**0**	**0**	**0**
Jun. 24, 2021	Rabies vaccination with a 2-dose PrEP schedule for certain individuals	Rabies	IMOVAX RabAvert	**0**	**N/A**	**0**	**0**	**0**	**0**	**0**
Jun. 25, 2021	PCV15 use in adults aged 65 years and older (PCV15 alone)	Pneumococcal Disease	Vaxneuvance	**0**	**0**	**0**	**0**	**0**	**0**	**0**
Jun. 25, 2021	PCV15 use in adults aged 65 years and older (PCV15+PPSV23)	Pneumococcal Disease	Vaxneuvance	**0**	**0**	**0**	**0**	**4**	**0**	**0**
Jun. 25, 2021	PCV20 Use Among Adults ≥65 Years Old	Pneumococcal Disease	Prevnar 20	**0**	**0**	**0**	**0**	**0**	**0**	**0**
Aug. 13, 2021	Use of an additional does of mRNA COVID-19 vaccine in immunocompromised people	COVID-19	Comirnaty SPIKEVAX	**0**	**0**	**0**	**0**	**0**	**0**	**0**
Sep. 29, 2021	Use of recombinant zoster vaccine in immunocompromised adults aged 19 years and older	Herpes Zoster/Shingles	SHINGRIX	**0**	**0**	**0**	**0**	**0**	**0**	**0**

Sep. 29, 2021	PCV15 use in adults aged 19-64 years	Pneumococcal Disease	Vaxneuvance	**0**	**0**	**0**	**0**	**4**	**0**	**0**
Sep. 29, 2021	PCV20 use in adults aged 19-64 years	Pneumococcal Disease	Prevnar 20	**0**	**0**	**0**	**0**	**0**	**0**	**0**
Sep. 29, 2021	Universal hepatitis B vaccination strategy in adults	Hepatitis B	Heplisav-B<br>Engerix-B<br>Recombivax HB	**0**	**0**	**0**	**0**	**1**	**0**	**0**
Sep. 29, 2021	Use of JYNNEOS (orthopoxvirus) vaccine primary series for healthcare personnel	Orthopoxviruses/Mpox	JYNNEOS	**0**	**N/A**	**0**	**0**	**0**	**0**	**0**
Sep. 29, 2021	Use of JYNNEOS (orthopoxvirus) vaccine primary series for research, clinical laboratory, and response team personnel	Orthopoxviruses/Mpox	JYNNEOS	**0**	**N/A**	**0**	**0**	**0**	**0**	**0**
Sep. 29, 2021	Use of JYNNEOS (orthopoxvirus) vaccine heterologous for those who received ACAM2000 primary series	Orthopoxviruses/Mpox	JYNNEOS	**0**	**N/A**	**4**	**0**	**0**	**0**	**0**
Oct. 20, 2021	Use of recombinant zoster vaccine in immunocompromised adults aged 19 years and older	Herpes Zoster/Shingles	SHINGRIX	**0**	**0**	**0**	**0**	**0**	**0**	**0**
Nov. 2, 2021	Initial EtR for use of Pfizer-BioNTech COVID-19 vaccine in individuals aged 5-11 years	COVID-19	Comirnaty	**4**	**0**	**0**	**0**	**0**	**0**	**0**
Jan. 12, 2022	Use of lyophilized CVD 103-HgR vaccine among children and adolescents aged 2-17 years	Cholera	VAXCHORA	**4**	**0**	**0**	**4**	**0**	**0**	**4**
Feb. 4, 2022	Use of Moderna COVID-19 vaccine primary series in individuals aged 18 years and older	COVID-19	SPIKEVAX	**0**	**0**	**0**	**0**	**0**	**0**	**0**
Feb. 23, 2022	Use of PreHevbrio hepatitis B vaccine in adults aged 18 years and older	Hepatitis B	PreHevbrio	**0**	**0**	**0**	**N/A**	**N/A**	**N/A**	**N/A**
Jun. 18, 2022	Use of Moderna COVID-19 vaccine in children ages 6 months-5 years and Pfizer-BioNTech COVID-19 vaccine in children ages 6 months-4 years under EUA	COVID-19	Comirnaty<br>SPIKEVAX	**0**	**0**	**0**	**4**	**0**	**0**	**0**
Jun. 22, 2022	Use of PRIORIX as an option for MMR prevention in individuals aged 6 months and older	MMR	PRIORIX	**0**	**0**	**0**	**0**	**0**	**0**	**0**

Jun. 22, 2022	Higher dose and adjuvanted influenza vaccines for persons aged 65 years and older	Influenza	Fluzone High-Dose Quadrivalent Fluad Quadrivalent	**0**	**0**	**0**	**0**	**0**	**0**	**0**
Jun. 22, 2022	PCV15 use in children aged 2-18 years with certain underlying medical conditions that increase the risk of pneumococcal disease	Pneumococcal Disease	Vaxneuvance	**0**	**0**	**0**	**1**	**0**	**0**	**0**
Jun. 22, 2022	PCV15 use in children aged younger than 2 years	Pneumococcal Disease	Vaxneuvance	**0**	**0**	**0**	**1**	**0**	**0**	**1**
Jun. 23, 2022	Use of Moderna COVID-19 vaccine in children ages 6-11 years and adolescents ages 12-17 years under EUA	COVID-19	SPIKEVAX	**0**	**0**	**0**	**4**	**0**	**0**	**0**
Jul. 19, 2022	Use of Novavax COVID-19 vaccine, adjuvanted in adults aged 18 years and older under EUA	COVID-19	Novavax COVID-19 Vaccine	**0**	**0**	**0**	**4**	**0**	**0**	**N/A**
Oct. 19, 2022	PCV20 use in adults aged 19 years and older who have previously received PCV13 only	Pneumococcal Disease	Prevnar 20	**1**	**0**	**0**	**0**	**0**	**0**	**1**
Oct. 19, 2022	PCV20 use in adults aged 65 years and older who have previously received both PCV13 and PPSV23	Pneumococcal Disease	Prevnar 20							
Oct. 19, 2022	PCV20 use in adults aged 19-64 years with an immunocompromising condition, cochlear implant, or cerebrospinal fluid leak who have previously received both PCV13 and PPSV23	Pneumococcal Disease	Prevnar 20	**1**	**0**	**0**	**0**	**0**	**0**	**1**
Feb. 22, 2023	Vaccination with JYNNEOS during Mpox outbreaks	Orthopoxviruses/Mpox	JYNNEOS	**0**	**0**	**0**	**0**	**0**	**0**	**4**
Feb. 23, 2023	Use of GSK adjuvanted RSVPreF3 vaccine (AREXVY) in adults Aged 60 years and older (65+)	RSV	AREXVY	**1**	**0**	**0**	**1**	**1**	**1**	**0**
Feb. 23, 2023	Use of GSK adjuvanted RSVPreF3 vaccine (AREXVY) in adults Aged 60 years and older (60-64)	RSV	AREXVY	**3**	**0**	**0**	**1**	**1**	**1**	**0**
Jun. 21, 2023	Use of Pfizer bivalent RSVpreF vaccine (ABRYSVO) in adults aged 60 years and older	RSV	ABRYSVO	**3**	**0**	**4**	**1**	**1**	**1**	**0**

Jun. 21, 2023	Safety of influenza vaccines for individuals with egg allergy	Influenza	Afluria Quadrivalent Fluarix Quadrivalent FluLaval Quadrivalent Fluzone Quadrivalent Fluzone High-Dose Quadrivalent Fluad Quadrivalent FluMist Quadrivalent	**0**	**0**	**N/A**	**4**	**1**	**0**	**0**
Jun. 22, 2023	PCV20 use in children aged younger than 2 years	Pneumococcal Disease	Prevnar 20	**0**	**0**	**1**	**0**	**0**	**0**	**0**
Jun. 22, 2023	PCV20 use in children aged 2-18 years with certain underlying conditions that increase the risk of pneumococcal disease	Pneumococcal Disease	Prevnar 20	**0**	**0**	**1**	**1**	**1**	**0**	**0**
Jun. 22, 2023	Use of Pfizer RSVpreF in pregnant people	RSV	ABRYSVO	**0**	**0**	**4**	**0**	**1**	**0**	**0**
Aug. 3, 2023	Use of Nirsevimab in children aged 8-19 months of age at increased risk of severe disease entering their second RSV season	RSV	Beyfortus	**0**	**0**	**0**	**0**	**1**	**0**	**1**
Aug. 3, 2023	Use of Nirsevimab in infants younger than 8 months of age born during the RSV season or entering their first RSV season	RSV	Beyfortus	**0**	**0**	**0**	**0**	**1**	**0**	**1**
Oct. 25, 2023	Vaccination with JYNNEOS for persons at risk of Mpox	Orthopoxviruses/Mpox	JYNNEOS	**0**	**0**	**0**	**0**	**0**	**4**	**0**
Oct. 25, 2023	Use of Pfizer MenABCWY vaccine when MenACWY and MenB are indicated in the same visit for healthy individuals aged 16-23 years and at-risk individuals aged 10 years and older	Meningococcal disease ABCWY	Penbraya	**4**	**0**	**0**	**0**	**1**	**1**	**1**

[1] Variability was measured on a scale from 0 (none) to 4 (very high).
